# Treatments for Recurrent Aphthous Stomatitis: A Literature Review

**DOI:** 10.3390/dj13020066

**Published:** 2025-01-31

**Authors:** Maurizio D’Amario, Giordano Foffo, Filippo Grilli, Mario Capogreco, Tommaso Pizzolante, Sofia Rastelli

**Affiliations:** Department of Life, Health and Environmental Sciences, University of L’Aquila, 67100 L’Aquila, Italymario.capogreco@univaq.it (M.C.);

**Keywords:** aphthous stomatitis, laser therapy, therapeutics, SAIDs, pain, healing

## Abstract

Background/Objectives: This literature review aims to provide guidance on the treatment of recurrent aphthous stomatitis (RAS) based on studies published over the past 10 years. Methods: This study included randomized clinical trials involving human patients from 2013 and 2023, published in any language. The trials examined both pharmacological and non-pharmacological treatments for aphthous lesions, mainly focusing on the adult population, with pain management as the primary outcome. The research was conducted using PubMed, EMBASE, and CINHAL databases. Results: Most of the 45 analyzed studies focused on non-pharmacological therapies, which led to positive results with minimal adverse effects or contraindications, even when compared to cortisone-based treatments. Laser therapy also showed excellent results, particularly in the immediate post-treatment period. Non-pharmacological therapies appeared to offer the best risk–benefit ratio for patients suffering from RAS. Conclusions: Treatment should be individualized based on the patient’s specific form of RAS, and laser therapy can be used either as a standalone treatment or as an adjunct to other treatments considered in the review.

## 1. Introduction

Recurrent aphthous stomatitis (RAS) is one of the most common conditions affecting the oral mucosa, with an average prevalence of 20% in the population [1,2,3]. It is characterized by recurring ulcers in the oral cavity that can persist for several years [3].

The destruction of the mucosa is driven by a T cell-mediated immune response, leading to the production of tumor necrosis factor-alpha (TNF-α), a key inflammatory mediator. This, in turn, triggers cytotoxic T cells (CD8+) which destroy epithelial cells [4]. Although the pathogenetic mechanism is well understood, the precise etiology of RAS lesions remains unknown. Several factors have been proposed as potential triggers [2], including:

- Allergies;
- Genetic predisposition;
- Hematological deficiencies;
- Hormonal imbalances;
- Immunological factors;
- Infectious agents;
- Nutritional deficiencies;
- Smoking cessation;
- Physical or mental stress;
- Trauma.

Each of these agents can initiate an immune reaction, resulting in the destruction of epithelial cells and the formation of characteristic ulcers. Additionally, systemic diseases such as Behçet’s disease, can present as RAS [2,3,4,5].

There are three clinical variants of RAS, distinguished by the size, number, and duration of the lesions [2,3]:

1. Minor aphthous ulcers: These are the most common form, typically presenting with one to five lesions, each 3–10 mm in size, round or oval, with a gray/yellow base, and erythematous edges. They often affect non-keratinized mucosa, cause significant pain, and usually heal within 7–10 days.
2. Major aphthous ulcers: These ulcers are larger (1–3 cm in diameter), deeper, and take longer to heal (2–6 weeks). They commonly affect the labial mucosa, soft palate, and tonsillar surfaces, and account for 10% of RAS cases. Healing may leave scarring.
3. Herpetiform ulcers: This variant involves a much higher number of smaller lesions (1–3 mm), with up to 100 ulcers at once. These ulcers can merge into larger lesions and primarily affect non-keratinized, non-adherent mucosal surfaces. They typically heal within 7–10 days.

Regardless of the form, RAS lesions are highly painful, often disproportionately so in relation to their size. This review aims to provide guidance on the treatment of aphthous lesions, whether idiopathic or related to systemic diseases, based on studies conducted over the past 10 years. It will examine both pharmacological and non-pharmacological treatments for RAS in adults, comparing outcomes with those of placebo or untreated groups [5]. The primary focus will be on pain management during ulcer healing, with the goal of identifying the most effective treatment methods based on recent research. The literature showed numerous clinical trials published in the last 10 years but found only a few systematic reviews conducted over 5 years of trials. This review aims to analyze a broader range of studies on RAS treatment, providing a more robust picture of injury management. The analysis included pharmacological and homeopathic interventions, comparing their effectiveness with placebo or alternative treatments. Results will be evaluated based on the quality of disease management, particularly in terms of pain control.

## 2. Materials and Methods

### 2.1. Protocol and Registration

The PRISMA statement was taken as a reference to structure the following literature review; the registration of the protocol was done through the PROSPERO platform which assigned the following registration number: CRD42023382368.

### 2.2. Eligibility Criteria

For the eligibility of studies, only randomized clinical trials which met the following criteria were included:

- Studies published in the last 10 years: the decision to analyze 10 years of clinical trials was made to give more publications to support the study review;
- Studies written in any language;
- Studies conducted on human patients (of any gender);
- Studies on patients suffering from recurrent aphthous disease;
- Studies in which a therapy is applied to improve the course of the aphthous pathology or/and to speed up its recovery.

Studies in which a comparison therapy such as a placebo or other active substance was applied were included.

The study exclusion criteria included the following:

- Case report studies;
- Case series studies;
- Observational studies;
- Prospective and retrospective studies;
- Reviews;
- Studies not conducted on human patients;
- Studies unrelated to RAS, such as those addressing lesions secondary to other diseases (Crohn’s disease, Behcet’s disease);
- Studies involving polytherapy or therapies affecting ulcer healing;
- Studies proposing adjuvant treatments as part of a broader therapeutic regimen.

### 2.3. Sources of Information

Searches were conducted using PubMed, EMBASE, and CINHAL databases. Only studies published within the past 10 years (up to January 2023) were included.

### 2.4. Research and Selection of Studies

Randomized clinical trial publications from the past 10 years (2013–2023) were considered for this review. These studies were conducted on human patients and published in any language. The following key terms were used in different combinations: “treatment”, “recurrent aphthous stomatitis”, “RAS”, “cortisone”, “laser”, “clorexidine”, “complementary therapies”. Boolean operators such as AND, OR, and NOT were applied to refine the search results.

Studies proposing any type of treatment for managing canker sores were included, while studies addressing therapies for primary diseases where RAS was a secondary effect were excluded. Relevant studies—those where the title and content aligned with the topic and were fully accessible to the team—were selected. Studies with incongruent titles or content, were discarded.

The search yielded a total of 123 studies on the subject. After eliminating 28 duplicates, 95 studies were left for further evaluation by title and abstract. Of these, 47 were excluded for not focusing on RAS treatment or pain management.

Three additional studies were excluded based on the exclusion criteria: they involved aphthous lesions as part of a complex pathological condition, such as Crohn’s disease or Behçet’s disease, where a primary disease was treated with oral ulcers as a secondary effect; patients were undergoing polytherapy or single therapy for other conditions, where the drugs affected the healing of oral ulcers; or the proposed treatment was an adjuvant to a more complex therapy involving multiple agents.

**Figure 1:**
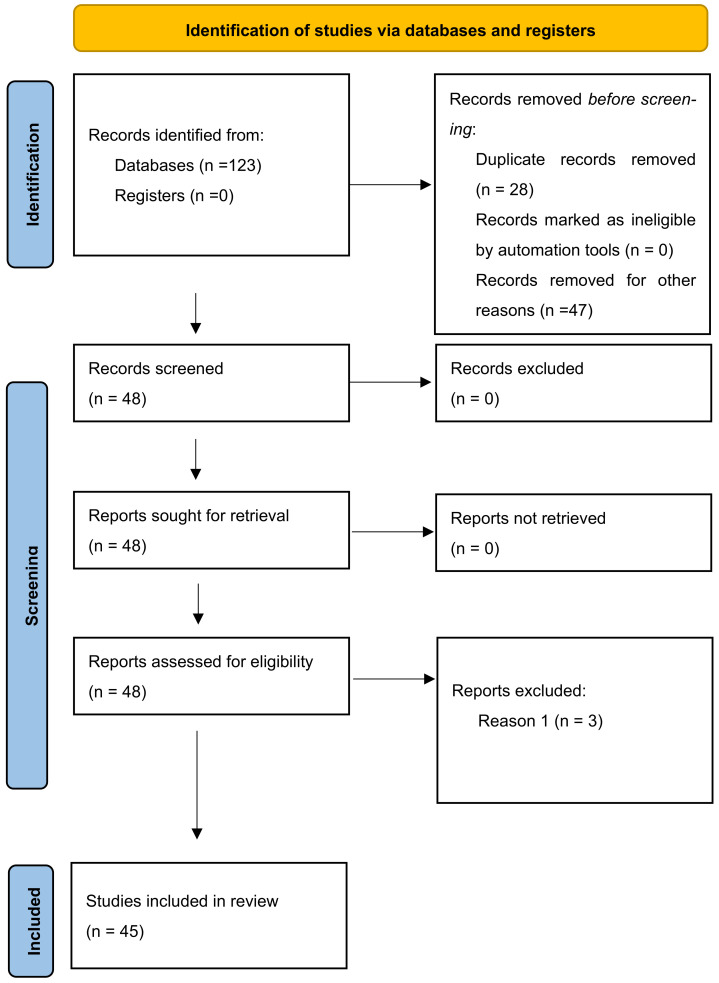
Flow chart of the PRISMA review process steps.

In conclusion, 45 studies met the eligibility criteria and were fully evaluated (Figure 1) [6,7,8,9,10,11,12,13,14,15,16,17,18,19,20,21,22,23,24,25,26,27,28,29,30,31,32,33,34,35,36,37,38,39,40,41,42,43,44,45,46,47,48,49,50].

### 2.5. Data Extraction

Data extraction was independently conducted by two authors, who assessed key aspects of each trial, including the first author and publication date, study design, sample size, type of intervention and comparison (placebo or alternative therapy), the pathological variant of the disease, the method of therapy administration, and outcomes. These details were compiled into a comprehensive table (Table 1), with each study represented in a row and the extracted data organized into columns. This table was used to facilitate comparisons between the studies, enabling us to generate meaningful results related to the study’s primary question (Table 1). The results were analyzed separately for single-blind and double-blind trials, with final considerations drawn to highlight distinctions between the two study designs.

### 2.6. Risk of Bias in Studies

**Figure 2:**
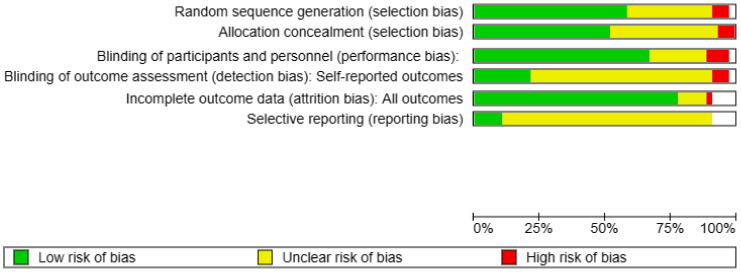

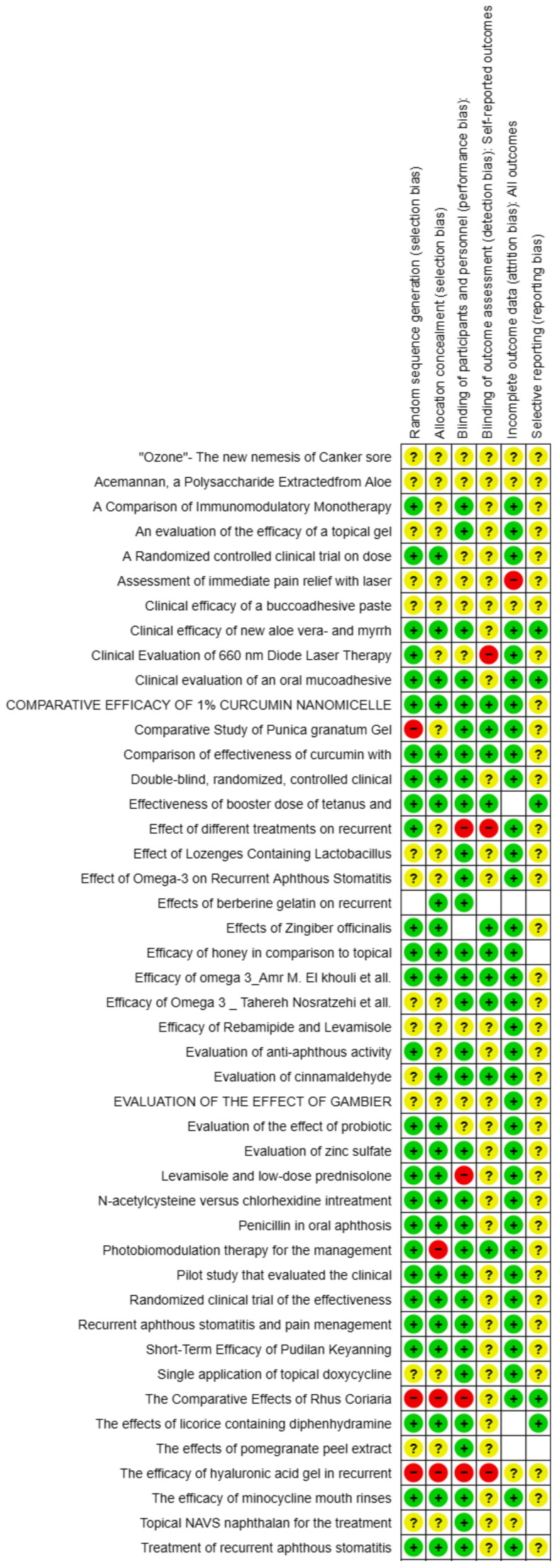
Schematic diagram summarizing the risk assessment of bias in each study.

Two authors independently assessed the risk of bias by evaluating the key ones in each included study: selection bias, performance bias, detection bias, attrition bias, and reporting bias. The “Cochrane RevMan” v 5.4 tool was used to develop the following diagrams (Figure 2).

## 3. Results

### 3.1. Risk of Bias

Regarding the risk analysis of bias in studies, the risk assessment showed a low risk for selection bias, performance bias, and attrition bias. However, for detection bias and reporting bias, the results showed a level of risk that was often unclear. A high risk of bias was detected in a few studies but limited to certain parameters. Overall, the clinical trials examined were with a reduced risk of bias. Considering the large number of articles referenced in this review, we focused on presenting the most relevant studies in the results, all of which are further discussed in the following section.

### 3.2. Topical Treatments

Bhalang K. et al. [9] investigated the use of acemannan, an extract of Aloe vera, and found that, in terms of pain relief assessed on specific days, acemannan achieved an average score of 7.5. In the control group, the mean score was 6.5, while the corticosteroid group had an average satisfaction score of 8.3.

In a study by Mansour G. et al. [13], 0.5% extracts of myrrh and Aloe vera were used to create new mucoadhesive oral gels. The study analyzed ulcer size, pain, erythema, and exudation on days 4 and 6. Among the patients treated with the Aloe vera gel, 76.6% showed complete ulcer healing; 86.7% and 80% showed the disappearance of erythema and exudation, respectively, particularly by day 6. Additionally, 76.7% of the patients treated with myrrh reported almost no pain on day 6.

Bakhshi M. et al. [16] studied the effects of a 1% curcumin nanomicelle gel and 2% plant-based curcumin gel on 48 patients. Pain scores, measured by the VAS scale, were 3.75 ± 0.9 in the curcumin gel group and 2.58 ± 0.93 in the nanomicelle group on day 4, indicating a statistically significant difference. Pain scores for both groups reached zero by day 7.

In the study by Deshmukh R.A. et al. [18], curcumin was compared to triamcinolone acetonide (a corticosteroid drug) for treating RAS. No statistically significant difference was observed between the two groups. The VAS scores on day 4 were 0.4 ± 0.67 in the experimental group and 0.5 ± 1.07 in the control group; on day 5, the scores were 0.06 ± 0.20 and 0.2 ± 0.61, respectively, and both groups scored zero on day 7.

A randomized clinical trial with 125 patients evaluated four products (silver nitrate, propolis, rhubarb extract, and walnut extract) as complementary therapies against a placebo. This study demonstrated statistically significant symptom resolution, particularly in the group treated with silver nitrate (mean symptom resolution time of 1.16 days). However, no VAS values were reported [40].

Ofluoglu D. et al. [10] tested a glycerol oxide gel (Triester) as an alternative to 0.1% triamcinolone acetonide (a corticosteroid), with a placebo group for comparison. The TGO group demonstrated a greater reduction in pain on days 2, 4, and 6, as well as a higher efficacy index and a significant reduction in lesion size on days 4 and 6.

### 3.3. Systemic Supplements

In a study by Hadian Z. et al. [21], an omega-3 supplement was administered, and the VAS scores after three months were 4.9 ± 0.96 in the test group and 7.2 ± 1.39 in the placebo group. After six months, the scores were 3.65 ± 1.78 and 7.2 ± 1.36, respectively, showing significantly better results for the test group at both intervals.

Nosratzehi T. et al. [27] also investigated an omega-3 polyunsaturated fatty acid supplement, with the placebo group recording a VAS score of 4.64 at six months compared to 3.04 in the test group.

Similarly, El Khouli A.M. et al. [28] used a systemic omega-3 supplement (1000 mg soft gelatin capsules). VAS scores in the placebo group were 6.76 ± 3.32 at two months, 6.40 ± 3.40 at four months, and 6.60 ± 4.31 at six months, compared to the test group’s scores of 5.80 ± 3.16, 3.04 ± 2.31, and 1.48 ± 2.31 at the same intervals.

### 3.4. Laser Treatments

Prasad and Pai A [11] conducted a study using a single session of CO_2_ laser treatment on lesions, compared with a placebo. Pain scores in the laser group dropped significantly from pretreatment (8.48 ± 0.71) to immediately after treatment (0.68 ± 0.6; *p* < 0.001), with better outcomes for healing time as well.

Soliman and Mostafaa [14] applied low-energy laser therapy with a 660 nm diode laser. The laser-treated group showed superior results across all fields, with VAS scores of 91.82 ± 18.34 for the experimental group and 8.00 ± 13.98 for the control group on day 4. By day 6, the efficacy index reached 100 in all study group cases, while the control group’s average was 48.00 ± 31.55.

Huo X. et al. [25] compared laser treatment against conventional pharmacological treatments for ulcerative lesions. The VAS score in the laser group was 2.04 ± 0.44 on day 1, significantly better than the pharmacological group (4.85 ± 0.40). On day 3, the scores were 0.60 ± 0.25 and 3.00 ± 0.44, respectively, favoring the laser group. By day 7, the results were close to zero and comparable in both groups.

Bardellini E. et al. [38] conducted a study on diode laser treatment in 60 children with MiRAS, assessing results on days 4, 7, and 10. A statistically significant difference was observed from the first day in the intervention group (VAS score at T1: 1 ± 0.72) compared to the placebo group (VAS score at T1: 3 ± 1.38, *p*-value = 0.0001).

Another randomized study assessed pain management using low-level laser therapy (wavelength 809 nm, power 60 mW, frequency 1800 Hz, duration 80 s per treatment, dose 6.3 J/cm^2^). VAS values dropped from 84.7 on day 0 to 31.5 on day 2, while the placebo group showed a minor change from 81.7 to 76.1 [41].

A study by Yilmaz H.G. et al. [50] examined the effects of Er,Cr:YSGG laser irradiation on pain reduction and healing speed in recurrent aphthous stomatitis. The VAS scores showed significant improvement in the intervention group, with the most notable difference immediately after treatment (*p* < 0.01) 

The effectiveness of various therapies for the treatment of recurrent aphthous stomatitis (RAS) and mucosal inflammatory recurrent aphthous stomatitis (MiRAS) were published in both single-blind and double-blind designs. A detailed examination of both types of trials allowed for a better understanding of the comparative benefits and limitations of the treatments assessed, while also providing a clearer insight into the potential biases inherent to different trial designs.

### 3.5. Single-Blind Studies

Among the single-blind studies, which involved patients being unaware of the assigned treatment but with researchers knowing the treatment allocation, several therapies were evaluated. For example, Dharmavaram A.T. et al. [6] studied the effects of ozonized oil, sesame oil, and placebo in a sample of 30 participants with RAS. The study demonstrated that ozonized oil was associated with a reduction in ulcer size, pain, and overall ulcer healing, while placebo showed a limited effect. Xue Y et al. [7] compared levamisole, prednisolone, and placebo in a larger cohort of 350 participants. The results indicated that both levamisole and prednisolone led to a significant reduction in ulcer number, size, and pain intensity compared to placebo, though the study’s single-blind design introduced the potential for observer bias in these results. Other single-blind studies also yielded promising results, though the potential for bias must be considered. For instance, Soliman and Mostafaa [14] found that diode laser therapy significantly reduced pain and ulcer size in a sample of 20 participants with MiRAS. Similarly, Sharda N. et al. [35] compared levamisole, levamisole combined with low-dose prednisolone, and placebo in 50 participants, observing improvements in ulcer pain, number, size, and the frequency of episodes. However, the limited blinding in these studies could have influenced the subjective outcome measures, such as pain intensity and healing time.

### 3.6. Double-Blind Studies

On the other hand, the double-blind studies, where both participants and researchers were unaware of treatment assignments, generally provided more reliable and robust data. A study by Bhalang K. et al. [9] with 180 participants investigated the effects of triamcinolone and acemannan in Carbopol compared to placebo in the treatment of RAS. The results showed that both triamcinolone and acemannan significantly reduced ulcer size, pain, and improved patient satisfaction, with the double-blind design minimizing bias in the reported outcomes. Similarly, Ofluoglu D et al. [10] in their study of 180 participants, comparing triamcinolone acetonide gel, TGO gel, and placebo, found that triamcinolone treatment notably reduced ulcer size and pain compared to placebo. In studies involving topical treatments, such as those by Pedersen AML et al. [29] and Hadian et al. [21], the double-blind design ensured unbiased results when comparing probiotics, omega-3 supplements, and placebo. In both studies, significant improvements were seen in ulcer healing, pain reduction, and the frequency of episodes, with omega-3 and probiotics outperforming the placebo groups. Other therapies evaluated in double-blind studies, such as curcumin nanomicelle gel (Bakhshi M. et al. [16]) and pomegranate flower gel (Tavangar et al. [17]), demonstrated significant reductions in ulcer size and pain compared to placebo, showcasing the efficacy of these novel treatments. Moreover, laser therapy, such as the one studied by Huo X. et al. [25] involving diode laser versus triamcinolone treatment, showed promising results in reducing pain intensity and promoting ulcer healing. In this study, both therapies were effective, but the double-blind design helped ensure that the observed effects were not influenced by bias.

When considering the various treatments evaluated, double-blind studies generally offered more reliable evidence due to their stronger control of biases, particularly observer bias and patient expectations. The double-blind design helped to prevent any influence from researchers or participants on the perceived outcomes, ensuring that the results more accurately reflected the true efficacy of the treatments being tested. In contrast, single-blind studies, while still valuable, were more susceptible to bias, especially in subjective measures such as pain and healing time, where the expectations of both participants and researchers could affect the results.

In conclusion, the comparison between single-blind and double-blind studies revealed that while both types of studies provided useful insights into the effectiveness of therapies for RAS and MiRAS, the double-blind design offered more robust and unbiased data, particularly when evaluating subjective outcomes. The therapies assessed in these studies, such as laser treatment, omega-3 supplements, and curcumin gels, showed promising results in reducing ulcer size, pain, and healing time, with the double-blind studies generally providing stronger evidence of their efficacy.

## 4. Discussion

Pain management was a common parameter across all studies included in this review and formed the primary focus of our concluding remarks. Although other parameters were assessed, they were often not comparable and prone to evaluation inaccuracies. Among the various treatments analyzed, low-level laser therapy (LLLT) emerged as a promising approach and will be discussed in greater detail in this section to highlight its potential as a key therapeutic option.

Among the 45 studies selected for this literature review, the most pertinent articles were discussed in both the results and discussion sections. Natural-origin materials were frequently utilized, though pharmacological evaluations were also conducted. Most studies employing natural compounds demonstrated notable results in terms of pain and lesion size. Several studies compared natural products with corticosteroid drugs, while others compared them with placebos, often with positive outcomes. The use of Aloe vera, curcumin, and propolis stood out with strong results, while compounds such as honey, pomegranate, ginger, fenugreek, and ozonated oil also showed favorable outcomes.

In the study by Bhalang K. et al. [9], an Aloe vera extract (Acemannan) was compared with a placebo and a corticosteroid-treated group, revealing positive results for Aloe vera against the placebo, although corticosteroids were more effective. Aloe vera proved to be a good alternative to pharmaceutical drugs.

In their study, Mansour G. et al. [13] treated patients with myrrh and Aloe vera. The mucoadhesive gels made from these extracts showed promising results, with myrrh performing better than Aloe vera.

Bakhshi M. et al. [16] also studied curcumin, applied as a gel in 1% and 2% concentrations. Both concentrations were effective for RAS treatment, with particularly positive results observed in the 1% curcumin nanomicelle group.

In a study by Dharmavaram A.T. et al. [6], ozonated oil outperformed the placebo group starting from day 4, though the small sample size limited the study’s generalizability.

Omega-3 supplements were evaluated in three long-term studies (6 months), all of which showed excellent results without side effects. These studies described similar benefits in reducing RAS recurrence. In Hadian Z. et al. study [21], omega-3 supplements were tested on RAS patients based on previous evidence of anti-inflammatory effects. The omega-3 group showed significant improvements in disease questionnaire scores and oral health-related quality of life after 3 and 6 months.

Nosratzehi T. et al. [27] similarly used an omega-3 polyunsaturated fatty acid supplement in a study involving 50 patients, divided into trial and placebo groups, with the trial group receiving 1000 mg omega-3 capsules. Although this study was limited to a placebo comparison, omega-3 proved to be an effective treatment.

El Khouli A.M. et al. [28] also administered 1000 mg omega-3 soft gelatin capsules daily over 6 months to 50 patients divided into placebo and test groups. The results were significantly better in the test group across all recorded parameters, supporting the findings of Hadian et al. [21] and Nosratzehi et al. [27].

Other plant-based compounds, such as ageratin [39], coriacea [44], punica [17], cinnamaldehyde [31], and tobacco leaves [30], were also studied. Chitosan [15], however, was the only naturally derived material that did not show satisfactory results.

In a study on triester glycerol oxide [10], the results surpassed those of the corticosteroid control group, partly due to its adhesive properties on mucosal surfaces, which highlighted the importance of formulation in treatment efficacy. Ofluoglu D et al. [10] studied this glycerol oxide gel (Triester) as an alternative to 0.1% triamcinolone acetonide in a placebo-controlled trial. 180 participants were divided into three groups, with measurements taken for ulcer size, pain (VAS scale), and efficacy index on days 0, 2, 4, and 6. Results across all parameters favored the TGO experimental group, suggesting it as a viable alternative to corticosteroids.

Multiple studies have examined laser therapy, all demonstrating statistically significant results [11,14,25,38,41,50]. The primary advantage of laser treatment was its effectiveness from the first day, a result also achievable with certain caustic chemicals, such as silver nitrate, which highlighted its potential when compared to other treatments, such as corticosteroids or placebo [40]. Laser therapy, especially low-level laser therapy (LLLT), has shown rapid pain relief and lesion size reduction, making it a promising treatment for recurrent aphthous stomatitis (RAS). The effectiveness of LLLT was consistently observed across various studies, showing no significant adverse effects and offering a non-invasive alternative to pharmaceutical treatments. These studies collectively underscored the role of laser therapy as one of the most promising approaches for RAS treatment.

The main limitations of this review was the risk of bias between studies because of the different number of patients and different age ranges, and the low amount of data related to systemic treatments and other local treatments recently reported.

## 5. Conclusions

While many of the compounds examined in this study were already obtainable, others are not yet suitable for oral treatments. The results of this review support the integration of low-level laser therapy (LLLT) as a viable treatment for RAS, especially considering its immediate effectiveness and minimal side effects.

For clinical use, the focus should be on products with validated formulations that govern reference trials. Treatment should be specific according to the patient’s RAS characteristics, with short-term treatments such as Aloe vera and hyaluronic acid in mucoadhesive formulas, recommended for minor and infrequent lesions. Long-term treatments such as omega-3 and probiotics, should be suggested for recurrent cases.

The application of laser therapy or silver nitrate can be effective for both forms of RAS, presenting good pain control. Laser therapy, in particular, offers a rapid onset of pain relief and can be considered for both acute and chronic RAS cases.

The future evolution of commercially available formulations for oral lesions will further amplify treatment options.

## Figures and Tables

**Table 1:** Data extraction, with the main results of the studies.

Author, Year	Study Design	Sample Size	Drug or Product/Comparison	Pathology Variant	Administration	Results
Dharmavaram AT et al., 2015 [6]	Randomized single-blinded	30	Ozonized oil/sesame oil/placebo	RAS	2 drops, 4 times/day for 5 day	Size, pain (VAS) and healing of ulcers.
Xue Y et al., 2018 [7]	Randomized single-blinded	350	Levamisole/prednisolone/placebo	RAS	Placebo: 1 tablet/day, 3 times/day for 6 months Levamisole: 1 tablet/day, 3 times/day for 6 months Prednisolone: 1 tablet/day, 3 times/day for 6 months	Number and size of ulcers. Pain intensity (NRS).
Deng Y et al. 2021 [8]	Randomized single-blinded	125	Prednisone/thalidomide	RAS	Prednisone: 15 mg/day for 7 days, 10 mg/d for next 3 days, 5 mg/day for next 3 days Thalidomide: Hide dose grope	Number of ulcers and pain intensity (VAS).
Bhalang K et al., 2013 [9]	Randomized double-blind study	180	Triamcinolone/0.5% acemannan in Carbopol/pure Carbopol	RAS	3 times/day for 7 days	Size of ulcers, pain (VAS), patient satisfaction.
Ofluoglu D et al., 2017 [10]	Randomized double-blinded study	180	0.1% triamcinolone acetonide gel/TGO gel/placebo gel	RAS	4 times/day for 7 days	Size and pain (VAS) of ulcers.
Prasad RS and Pai A 2013 [11]	Randomized single blinded	25	Carbon dioxide (CO_2_) laser/placebo	MiRAS	A single administration of therapy laser/placebo	Time of healing, size of ulcers and pain (VAS).
Ansari M et al., 2013 [12]	Randomized double-blinded study	60	Fenugreek mucoadhesive paste (FBP)/dexamethasone mouthwash	RAS	Three times daily on the lesion for 10 consecutive days	Pain intensity (VAS), size of ulcers, severity of erythema and exudate.
Mansour G et al., 2014 [13]	Randomized double-blinded	90	Aloe vera mucoadhesive gel/myrrh-based mucoadhesive gel/placebo	MiRAS	4 times/day for 5 days	Ulcer size, pain scale (VAS), erythema, and level of exudate
Soliman HA and Mostafaa D. 2019 [14]	Randomized single-blinded	20	diode laser treatment/placebo (diluted sodium bicarbonate)	MiRAS	Study group, single diode laser application; control group, placebo 4 times/day	Pain assessment of ulcers (VAS), assessment of ulcer size, The effectiveness indices (EI) assessment of healing, post-operative complications assessment
Shao Y and Zhou H. 2020 [15]	Randomized double-blind study	72	Test group: film containing chitosan Control Group: polyvinyl alcohol film	RAS	2 applications/day for 6 days	Pain score (VAS), adverse effects, ulcer size
Bakhshi M et al., 2022 [16]	Randomized double-blinded study	48	1% curcumin nanomicelle gel or 2% curcumin gel	RAS	3 times/day for 1 week	Pain of ulcers (VAS), size of lesions (millimeters)
Tavangar A et al., 2019 [17]	Randomized double-blinded study	60	Gel from pomegranate flower/triamcinolone muco-adhesive paste/placebo	MiRAS	3 times/day until the ulcer healed	Pain of ulcers (VAS), size of ulcers, complete healing
Deshmukh RA and Bagewadi AS. 2014 [18]	Randomized double-blinded study	60	Curcumin gel/Triamcinolone Acetonide gel	RAS	3 times/day for 7 days	Pain (VAS), size, and number of the ulcers
Jiang XW et al., 2013 [19]	Randomized double-blinded study	134	Paste A Diosmectite and basic fibroblast growth factor. Paste B primarily contained DS. Paste C primarily contained bFGF. Paste D placebo paste.	MiRAS	4 times/day for 5 days	Size, pain (VAS) of ulcers, adverse drugs effect.
Pedersen AML et al., 2019 [20]	Randomized double-blinded study	20	Probiotics lozenges (Lactobacillus reuteri)/placebo lozenges	RAS	Two lozenges for day (morning and evening) for 90 days	Number, size, duration, ulcer-free period, pain (VAS).
Hadian Z et al., 2021 [21]	Randomized double-blinded study	40	Omega-3 tablet/placebo	RAS	Three times/day, for 6 months	Number, duration of ulcers, ulcer frequency period, mucosal site, and VAS criteria for pain
Habibzadeh S et al., 2019 [22]	Randomized triple-blinded study	70	Diphtheria toxoids vaccine/Placebo	MiRAS	Intervention group: oral colchicine 1 mg/day for 5 days; intramuscular dose of 0.5 mL of Td vaccine. placebo group: oral colchicine 1 mg/day for 5 days; injection of single dose of 50 mg of vitamin B6	Number and size of aphthous ulcers, recovery time, intervals between episodes, pain intensity (VAS).
Du Q et al., 2018 [23]	Randomized double-blinded study	59	Dried ginger rhizome membrane (DGRM)/placebo membrane (PM)	RAS	A piece of membrane each time twice a day, until healing of ulcers	Levels of epidermal growth factor (EGF) and tumor necrosis factor (TNF)-a in saliva. Pain level (VAS); adverse reaction.
Jiang XW et al., 2013 [24]	Randomized double-blinded study	84	Gelatin containing berberine (5 mg/g)/Placebo gelatin	MiRAS	4 times/day, for 5 days	Pain level (VAS), size of ulcers, erythema and exudation
Huo X et al., 2020 [25]	Randomized single blinded	56	Diode laser group/Medication group (triamcinolone)	RAS	Laser group: irradiation time 20 s for 3 applications once daily for continuous 3 days/triamcinolone acetonide 0.1% 3 times/d until healing	Pain intensity (VAS), healing of the ulcer.
El-Haddad SA et al., 2014 [26]	Randomized single blinded	94	Group I: Commercial honey Group II: Triamcinilone ointment acetonide Group III: orabase which acts as a protective paste	RAS	3 times/day for 8 days	Size, healing, pain (VAS) and erythema of ulcers
Nosratzehi T et al., 2016 [27]	Randomized double-blind study	50	Omega-3 capsules 1000 mg/Placebo Group	RAS	3 times/day for 6 months	Pain (VAS), size, duration and recurrence of ulcers
El Khouli AM et al., 2014 [28]	Randomized double-blind study	50	Omega-3 soft capsules (1000 mg)/Placebo	RAS	Omega-3 (1 g) 3 times/d; placebo for 6 months	Number of new ulcers, duration of episodes, pain level (VAS)
Parvathi Devi MK et al., 2014 [29]	Pilot study	100	Levamisole/Rebamipide	RAS	50 mg, 3 times/day for 3 days, for 3 weeks/100 mg, 3 times/day for 1 week	Pain (0—no pain, 1—mild pain, 2—moderate pain, 3—severe pain), numbers, size and rate of ulcers
Vaziri S et al., 2016 [30]	Randomized double-blinded study	60	Decoction derived from the leaves of Nicotiana tabacum/placebo	MiRAS	10 mL 3 times/day for 5 days	Size and pain level of ulcers (VAS), safety assessment
Molania T et al., 2022 [31]	Randomized double-blinded study	44	Cinnamaldehyde/placebo	MiRAS	Three daily muco-adhesive patches in the morning, afternoon, and night for 7 days	Size and pain level of ulcers (VAS)
Dewi SRP et al.2020 [32]	Randomized single blinded	30	Gambier (Uncaria gambir)/placebo	RAS	Three times daily for seven days	Healing duration, changes in the size of the lesion, pain intensity (VAS)
Aggour RL et al. 2020 [33]	Randomized single blinded	120	*L. acidophilus* containing lozenges (ChocBalls)/Oracure oral gel (15 gm)	MiRAS	Lozenges twice daily, for 5 days	Size and pain level of ulcers (VAS)
Ghorbani A et al. 2020 [34]	Randomized double-blinded study	46	Zinc sulfate muco-adhesive tablet/placebo	RAS	Tablet for 7 days	Pain intensity (VAS), ulcer size, safety assessment
Sharda N et al. 2014 [35]	Randomized single blinded	50	levamisole (50 mg)/levamisole (50 mg) and low-dose prednisolone (5 mg)/placebo	RAS	Thrice daily for 3 consecutive days/week for 3 consecutive weeks	Pain (VAS) due to ulcers, number of ulcers/episodes, size of ulcers, duration of ulcers, and frequency of ulcers (episodes/month)
Halboub E et al. 2021 [36]	Randomized single blinded	54	N-acetyl cysteine (NAC)/0.12% chlorhexidine digluconate (CHX)	RAS	Mouthwash for 30s for 6 days	Pain intensity (VAS), time for healing
Owlia MB et al., 2020 [37]	Randomized double-blinded study	50	Topical penicillin powder/placebo	RAS	Four times/day for a week	Lesion size, severity of Pain (PRS), days to pain cessation, days to ulcer healing, and drug adverse
Bardellini E et al. 2020 [38]	Randomized single blinded	60	Photobiomodulation therapy (PBMT)/placebo	MiRAS	Laser therapy (diode laser, λ: 645 nm) for three consecutive days/same device without laser emission	Pain relief (VAS), lesion size reduction and parental satisfaction of the therapy.
Romero-Cerecero O et al. 2015 [39]	Randomized double-blinded study	56	Extract of *A. pichinchensis* at 5%/Triamcinolone at 0.1%	MiRAS	Three times daily until remission of the clinical condition, without exceeding a time period of 2 weeks	Pain intensity (VAS), clinical effectiveness, treatment adherence, therapeutic failure, and therapeutic success
Rodríguez-Archilla A et al. 2017 [40]	Randomized study	125	Silver nitrate/propolis/rhubarb/walnut/placebo	MiRAS	Apply/spray 3 times per day	Time for the disappearance of symptoms and lesions, adverse effect
Albrektson M et al. 2014 [41]	Randomized single blinded	40	Low-level laser therapy (LLLT)/placebo	RAS	Wavelength, 809 nm; power, 60 mW; pulse frequency, 1800 Hz; duration, 80 s for treatment on 3 occasions, with a 1-day interval/ same device without laser emission	Pain intensity (VAS), and patients experience of eating, drinking, and brushing teeth
Yang Y et al. 2016 [42]	Randomized double-blinded study	80	Pudilan Keyanning toothpaste (PKT)/placebo	MiRAS	Brush for 2–3 min, twice a day (in the morning and evening), each time covering two thirds the length of the toothbrush provided	Healing rate, healing period, pain (VAS), areas of the target ulcerated lesions, degree of exudation, and hyperemia
Vijayabala GS et al. 2013 [43]	Randomized single blinded	50	doxycycline hyclate/placebo	RAS	Topically applying the medicament or the placebo over the ulcer	pain intensity (VAS), number of ulcers, size of each ulcer and the duration
Lavaee F et al. 2022 [44]	Randomized single blinded	22	Rhus coriaria/triamcinolone	RAS	Three times/day for 6 days	Pain and size of the lesion (VAS)
Akbari N et al. 2020 [45]	Randomized double-blinded study	70	Diphenhydramine solution (DS)/di- phenhydramine-containing glycyrrhiza glabra (DSG)	RAS	Swish 3 mL of solution around mouth for about three minutes four times a day	Pain intensity (VAS), adverse effects
Darakhshan S et al., 2019 [46]	Randomized double-blinded study	56	Pomegranate peel extract (PPE)/placebo	RAS	Gel twice daily for one week	Ulcer size, pain (VAS) and healing duration
Koray M et al. 2016 [47]	Randomized single blinded	57	Topical hyaluronic acid gel (HA)/triamcinolone acetonide pomade (TA)	RAS	4 times per day for 7 days	Pain intensity (VAS)
Yarom N et al., 2017 [48]	Randomized double-blinded study	29	Minocycline mouthwash 0.2% and 0.5% solution	RAS	4 times a day, rinse mouth with 5 mL of the solution for at least 1 min	Pain intensity (VAS)
Rogulj AA et al., 2021 [49]	Randomized double-blinded study	27 + 30	Non-aromatic very rich in steranes (NAVS) naphthalan/0.05% betamethasone dipropionate	RAS/OLP	Three times daily for 4 weeks for OLP patients and three times daily for 7 days for RAS patients	Activity score (OLP patients), number of lesions, lesion diameter, pain intensity (VAS), impact of the disease on quality of life assessed by Oral health impact profile (OHIP)
Yilmaz HG et al., 2017 [50]	Randomized single blinded	40	Er,Cr:YSGG laser/placebo	MiRAS	Er,Cr:YSGG laser at an energy level of 0.25 W for 20 s/ same device without laser emission	Pain (VAS) Healing (HRAS)
